# Evaluation of the performance of QuantiFERON®-TB Gold plus test in active tuberculosis patients

**DOI:** 10.1016/j.jctube.2021.100223

**Published:** 2021-02-13

**Authors:** Cengiz Çavuşoğlu, Melike Yaşar-Duman, Mehmet Sezai Taşbakan, Meltem Işıkgöz-Taşbakan, Mehmet Nurullah Orman

**Affiliations:** a Department of Medical Microbiology, University of Ege, Izmir, Turkey; b Department of Chest Diseases, University of Ege, Izmir, Turkey; c Department of Infectious Diseases and Clinical Microbiology, University of Ege, Izmir, Turkey; d Department of Biostatistics and Medical Informatics, University of Ege, Izmir, Turkey

**Keywords:** QuantiFERON TB Gold Plus, Active TB, Spoligotyping, LTBI

## Abstract

The aim was to evaluate the sensitivity and the possible factors affecting the sensitivity of the QuantiFERON®-TB Gold Plus (QFT-Plus) assay in culture-positive active TB (Tuberculosis) patients, to investigate the possible causes of negative and indeterminate results in active TB patients, and to compare the QFT-Plus results of active TB patients and latent tuberculosis infection (LTBI) cases. The QFT-Plus assay was performed in 46 active TB patients and 64 LTBI. The sensitivity of the test was found as 79.5% in all culture-positive patients, 72.7% in the immunocompromised patients, and 86.4% in the non-immunocompromised patients. Compared to active TB, individuals with LTBI had a lower T-cell response and lower IFN-ɣ concentrations. It was determined that the immunocompromisation reduced the sensitivity of the test and the secreted IFN-ɣ concentrations and increased the indeterminate results in patients with active TB. There was no difference in secreted IFN-ɣ concentrations between M. tuberculosis clones, but higher IFN-ɣ concentrations in patients infected with M. tuberculosis strains compared to patients infected with zoonotic strains. Compared with active TB, response to “only to TB2” was significantly higher in LTBI. In conclusion, it was concluded that TB2 tube increased sensitivity in LTBI but may not contribute to sensitivity in active TB.

## 1 Introduction

It is estimated that approximately 1/4 of the world's population is latently infected with *Mycobacterium tuberculosis*. Individuals with latent tuberculosis infection (LTBI) have a 5–10% lifetime risk of developing active tuberculosis (TB), and most cases occur within the first 5 years after infection [1], [2], [3], [4]. According to the data of the World Health Organization (WHO) (https://www.who.int/tb/publications/global_report/en/), it is reported that there are approximately 10 million new TB cases in the world in 2018, and a total of 2 million people died due to TB, of which 1.2 million are HIV negative and 251.000 HIV positive. TB incidence in Turkey in 2017 was 14.6/100.000, and total number of 12.046 new TB cases were detected, including 66.1% pulmonary TB (PTB) and 33.9% extrapulmonary TB (EPTB) [5]. Treatment of active TB solely is not sufficient in the elimination of the disease, since LTBI is a source for new active TB patients [6]. For this reason, WHO recommends the diagnosis and prophylactic treatment of LTBI in individuals at risk for active TB development in high and middle-income countries [7].

There is no gold standard in the diagnosis of LTBI. However, interferon-gamma release assays (IGRA) such as QuantiFERON®-TB Gold in Tube (QFT-GIT) have been widely used in the diagnosis of *M. tuberculosis* infection in recent years [8]. Recently, some modifications were made in the QuantiFERON®-TB Gold in Tube (QFT-GIT) assay, and the QuantiFERON®-TB Gold Plus [QFT-Plus] (Qiagen, Hilden, Germany) was offered for use. The interferon-gamma (IFN-ɣ) released from peripheral blood mononuclear cells stimulated with ESAT-6 and CFP-10 antigens encoded by the region of difference-1 (RD1) of the *M. tuberculosis* genome is determined in the QFT-Plus assay. There are two TB antigen tubes (TB1 and TB2) in the QFT-Plus, containing ESAT-6 and CFP-10 peptide antigens. The QFT-Plus TB1 tube contains relatively long peptides that stimulate CD4+ T cells, while the TB2 tube contains an antigen cocktail consisting of short and long ESAT-6 and CFP-10 peptides to ensure the release of IFN-ɣ from CD8+ T cell as well as CD4+ T cells. CD8+ T cells are important components of host immunity for the control of *M. tuberculosis* infection. It is reported that significantly higher cytotoxic CD8+ T cell responses are observed in smear-positive and active PTB patients compared to smear-negative and LTBI patients. Based on these findings, the addition of peptides to stimulate CD8+ T cells is expected to increase the sensitivity of the test in detecting LTBI and active TB infection in patients with destroyed CD4+ T cells [10], [11].

In the present study, it was aimed to evaluate the sensitivity and the possible factors affecting the sensitivity of the QFT-Plus assay in culture-positive active TB patients, to investigate the possible causes of negative and indeterminate results in active TB patients, and to compare the QFT-Plus results of active TB patients and LTBI cases.

## 2 Materials and methods

### 2.1 Active TB patients and LTBI group included in the study

The study included 46 active TB patients with positive cultures for the *M. tuberculosis* complex, and 64 LTBI with lymphocyte count within normal limits, without immunosuppressive therapy, and without accompanying immunosuppression admitted to the Ege University Medical Faculty Hospital between January 2016 and December 2019. Demographic and clinical data and laboratory and culture results of patients were obtained from the Mycobacteriology Laboratory and hospital database.

### 2.2 QFT-Plus assay

The QFT-Plus assay was performed according to the manufacturer's recommendations. One ml of venous blood was collected from the patients into the Nile Control, Mitogen Control, TB1 Antigen, and TB2 Antigen tubes in the kit. Tubes were incubated at 37 °C for 16–24 h within 16 h after collection. After incubation, tubes were centrifuged at 3000×*g* for 15 min to separate plasma and were stored in the refrigerator at+ 4 °C for 0–4 days. 50 µl of freshly prepared working conjugate was added to all ELISA wells. Afterward, 50 µl plasma samples were added to Nile, TB1 Antigen, TB2 Antigen, and Mitogen wells and incubated for two hours at room temperature. After washing, 100 µl of enzyme substrate solution was added to the wells and incubated for 30 min at room temperature. 50 µl of stop solution was added to all wells and the optical density of each well was measured by reading in the ELISA reader. The analysis of the optical density values was carried out using the “QuantiFERON-TB Gold Analysis Software”. The results of the analysis were evaluated as positive, negative, or indeterminate. A positive test result was defined as antigen − nil ≥ 0.35 IU/mL and ≥25% of the nil sample, whereas a negative test was defined as antigen–nil < 0.35 IU/mL or <25% of nil when mitogen ≥ 0.5 IU/mL. The results were considered indeterminate if 1) nil > 8 IU/mL or 2) antigen–nil ≥ 0.35 IU/mL and < 25% of nil when the nil was ≤8.0 IU/mL and the mitogen response was <0.5 IU/mL. The IFN-ɣ value of ≥0.35 IU / ml antigen tube corrected with Nile was evaluated as positive [12].

### 2.3 Microbiological methods

The auramine-rhodamine fluorescent staining method was used for microscopic examination, and automated BACTEC MGIT 960 (BD, USA) culture system was used for mycobacterial culture. The spoligotyping method (Spoligotyping Kit; IsogenLifeScience, The Netherlands) was used for the genotyping and identification of the isolates grown in culture.

### 2.4 Statistical analysis

Statistical analysis was performed using IBM-SPSS 25.0 package program. Cross tables were created for categorical variables and chi-square analyzes were performed. Whether numeric variables are suitable for normal distribution was checked by the Shapiro-Wilk test. Comparison of two groups for variables without normal distribution was made by Mann-Whitney *U* test. Categorical variables were summarized as numbers and %, numerical variables as median (min., Max.). Statistical significance was accepted as p < 0.05.

## 3 Results

### 3.1 Active TB patients and LTBI included in the study

The QFT-Plus results of a total of 46 culture-positive active TB patients (27 males and 19 females) with a median age of 55 (19–83 years) and a total of 64 LTBI cases (45 males and 19 females) with a median age of 59 (22–91 years) were evaluated. Twenty four of 46 TB patients were immunocompromised, solid organ transplantation in 10, hematological malignancy in five, chronic kidney failure in three, HIV positive in two, steroid use in two, chronic liver failure in one, and lymphopenia in one. Five of the patients died during the follow-up period.

### 3.2 QFT-Plus results in active TB patients and LTBI

The QFT-Plus was positive in 35 (76.1%) of the TB patients included in the study, while nine (19.6%) patients had negative results and two (4.3%) patients had indeterminate results. Excluding the indeterminate results, the sensitivity of the test was found as 79.5% in all culture-positive patients (77.3% in the TB1 tube, 75% in the TB2 tube), 72.7% (16/22) in the immunocompromised patients (ICP), and 86.4% (19/22) in the non-immunocompromised patients (NICP) (chi-square; p = 0.262). The sensitivity of the test was determined as 69.2% (9/13) in smear-positive patients, 83.9% (26/31) in smear-negative patients (Fisher exact test, p = 0.414), 70.6% (12/17) in PTB, 82.6% (19/23) in EPTB (chi-square p = 0.478). Of the 35 active TB patients, 34 (97.1%) were positive in the TB1 tube and 33 (94.3%) in the TB2 tube, and of the 64 LTBI, 52 (81.3%) were positive in the TB1 tube and 57 (89.1%) in the TB2 tube. The relationship between the clinical and laboratory findings of patients and the results of QFT-Plus is summarized in Table 1, the comparison of positive QFT-Plus results in TB patients and LTBI is summarized in Table 2.

**Table 1:** QFT-Plus results according to the clinical and laboratory findings of the patients (n = 46).

OFT-Plus results	Involved organ				Microscopy		Immuno-compromisation	
	PTB		EPTB	Miliary	AFB^1^ +	AFB−	Yes	No
	Cavitary	Noncavitary						
Positive	3	9	19	4	9	26	16	19
Negative	2	3	4	–	4	5	6	3
Indeterminate	–	–	1	1	–	2	2	–
Total	5	12	24	5	13	33	24	22

1 AFB: Acid-Fast Bacillus.

**Table 2:** Comparison of positive QFT-Plus results in active TB patients and LTBI.

	QFT-Plus results (n, %)						
	TB1 + TB2 positive	P	TB1 positive	P	TB2 positive	P	Total
Active TB	32 (91.4%)	0.016	2 (5.7%)	0.38	1 (2.9%)	0.025	35(100%)
LTBI	45(70.3%)		7 (10.9%)		12(18.8%)		64(100%)

### 3.3 Quantitative QFT-Plus responses to TB antigen in active TB patients and LTBI

**Fig. 1:**
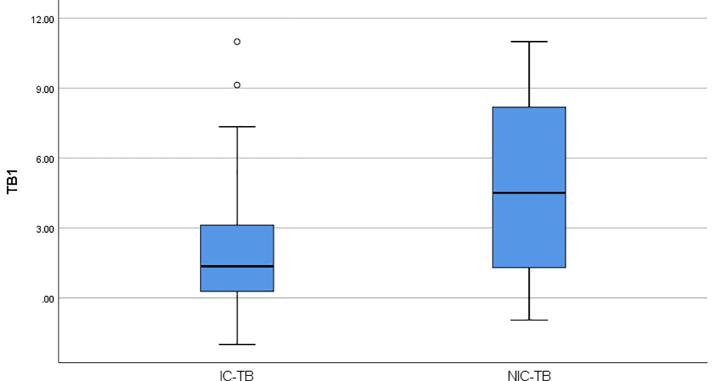

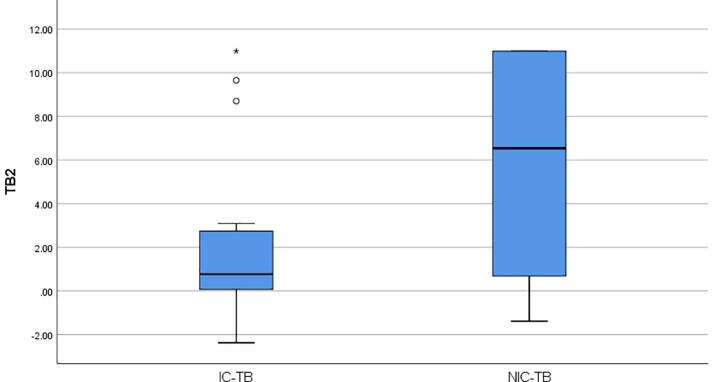
**a.** The median IFN-ɣ concentrations released in TB1 tubes of ICP and NICP. **b.** The median IFN-ɣ concentrations released in TB2 tubes of ICP and NICP.

The median IFN-ɣ concentration was 3.33 IU/ml in TB1 tube and was 2.89 IU/ml in TB2 tube, respectively in active TB patients (Wilcoxon Signed Ranks Test, p = 0.773). To evaluate the effect of immunosuppression on concentrations of IFN-γ released in QFT-Plus assay, active TB patients were separated into two groups as ICP and NICP. The median IFN-ɣ concentration (1.36 IU/ml) released in the TB1 tube was higher than the median IFN-ɣ concentration (0.77 IU/ml) released in the TB2 tube in 22 ICP (Wilcoxon Signed Ranks Test, p = 0.233), and the median IFN-ɣ concentration (6.54 IU/ml) released in theTB2 tube was higher than the median IFN-ɣ concentration (4.51 IU/ml) released in the TB1 tube in 22 NICP (Wilcoxon Signed Ranks Test, p = 0.136). In addition, the median IFN-ɣ values released in both TB1 and TB2 tubes (4.51 IU/ml to 1.36 IU/ml in TB1 tube, Mann-Whitney U, p = 0.038; 6.54 IU/ml to 0.77 IU/ml in TB2 tube, Mann-Whitney U, p = 0.031) and in mitogen tubes (>10 IU ml to 5.37 IU/ml, Mann-Whitney U, p = 0.022) were found to be higher in NICP than ICP. The median IFN-ɣ concentrations released in TB1 and TB2 tubes of ICP and NICP were shown in Fig. 1a and Fig. 1b.

**Fig. 2:**
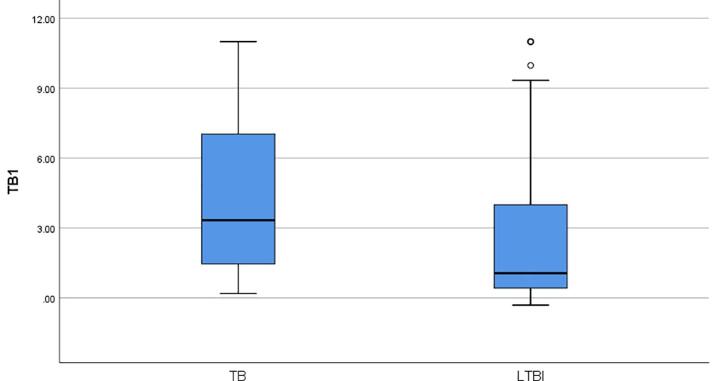

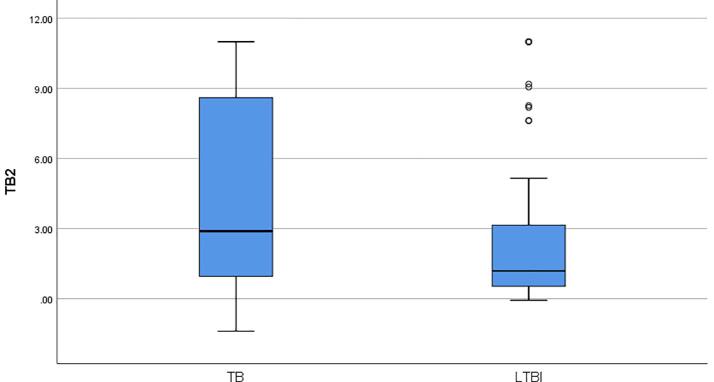
**a.** The median IFN-ɣ concentrations released in TB1 tubes of QFT-Plus positive-TB patients and LTBI cases**b.** The median IFN-ɣ concentrations released in TB2 tubes of QFT-Plus positive-TB patients and LTBI cases.

The median IFN-ɣ value (1.19 IU/ml) released in the TB2 tube was found to be higher than the median IFN-ɣ value (1.06 IU/ml) released in the TB1 tube in 64 LTBI (p = 0.745). In addition, compared with the LTBI, QFT-Plus positive TB patients were found to have higher concentrations of IFN-ɣ released in both TB1 (3.33 IU/ml to 1.06 IU/ml; Mann-Whitney U, p = 0.02) and TB2 tube (2.89 IU/ml to 1.19 IU/ml; Mann-Whitney U, p = 0.017). The median IFN-ɣ concentrations released in TB1 and TB2 tubes of TB patients and LTBI cases were summarized in Fig. 2a and Fig. 2b.

### 3.4 Quantitative QFT-Plus responses to TB antigen in *Mycobacterium tuberculosis* genotypes

**Fig. 3:**
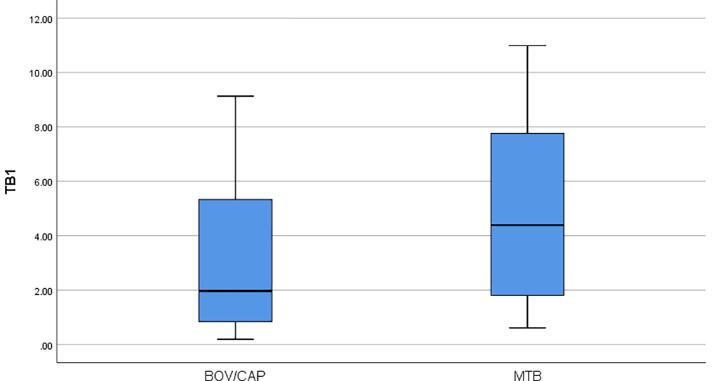

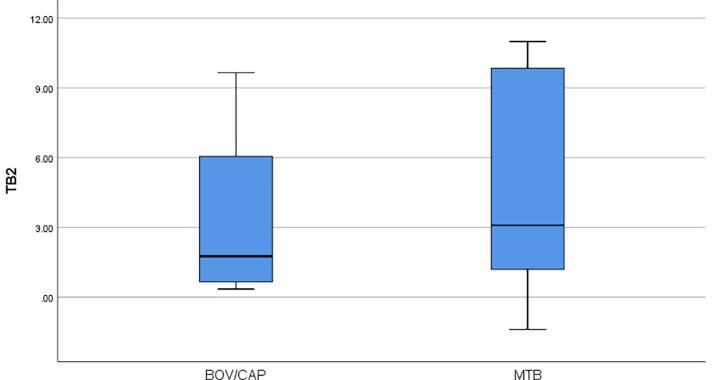
**a.** The median IFN-ɣ concentrations released in TB1 of QFT-Plus positive *M. tuberculosis* and *M. bovis/caprae* cases **b.** The median IFN-ɣ concentrations released in TB2 of QFT-Plus positive *M. tuberculosis* and *M. bovis/caprae* cases.

Mycobacterial cultures were positive for *M. tuberculosis* in 37 of 42 patients, *M. bovis* in 7 patients, and *M. caprae* in 2 patients. The QFT-Plus was positive in 77.1% (27/35) of patients growing *M. tuberculosis*, and 88.9% (8/9) of patients growing *M. bovis* or *M. caprae*. Of the 37 *M. tuberculosis* strains that were carried out spoligotyping, 11 were determined to be T family, five were H family, two were LAM7TUR family and 19 were from other families. There was no significant relationship between *M. tuberculosis* genotypes and concentration of IFN-ɣ released in QFT-Plus. The median IFN-ɣ values ​​released in the TB1 tube were found to be 4.39 IU/ml for *M. tuberculosis*-growing patients and 1.97 IU / ml for *M. bovis*/*caprae*-growing patients (Mann-Whitney U, p = 0.168 (0.179), and the median IFN-ɣ values released in the TB2 tube were 3.09 IU/ml for *M. tuberculosis*-growing patients and 1.76 IU/ml for *M. bovis*/*caprae*-growing patients (Mann-Whitney U, p = 0.237 (0.252). Of the *M. tuberculosis*-growing patients, 6 in TB1 tubes and 7 in TB2 tubes were detected > 10 IU/ml IFN-ɣ concentrations, whereas in *M. bovis/caprae*-growing patients were not detected > 10 IU/ml IFN-ɣ concentrations (Fig. 3a and Fig. 3b).

## 4 Discussion

When two indeterminate results were excluded, the sensitivity of QFT-Plus was 79.5% (35/44) in all culture-positive TB patients, 72.7% (16/22) in IC-TB, 86.4% in NIC-TB (19/22), respectively. However, the difference between the two groups was not statistically significant. It has been reported that malignancy, lymphopenia, and immunosuppression may be risk factors for false negative IGRA results in patients with TB infection and IGRAs show decreased sensitivity in ICPs [13], [14], [15], [16], [17]. Different results were obtained in studies investigating the sensitivity of QFT-Plus in active TB patients ranging between 86.5% and 100% in NICP [18], [19], [20], [21].

In our study, two patients (4.3%) with undetermined results had lymphopenia (260/mm^3^ and 860/mm^3^) and renal transplantation. In addition to technical errors in sample handling and processing, it is known that patients with low lymphocyte count, hematological malignancy/immunodeficiency, HIV and severe TB patients, and patients under immunosuppressive therapy have higher indeterminate results due to insufficient mitogen response [12], [22], [23]. In other studies, indeterminate result rates in QFT-Plus were found to be 4.6% in HIV-TB and 3.1–3.8% in NICPs [18], [19].

Various studies have reported that there is a positive correlation between *M. tuberculosis* load and CD8+ T cell response and the addition of CD8+ T cell stimulating antigens may increase the amount of IFN-ɣ compared to the situation where only CD4+ T cell stimulating antigens are present. It has been stated that smear-positive TB and clinically/radiologically severe PTB had a stronger CD8+ T cell response and higher IFN-ɣ values ​​secreted from the TB2 tube, compared with smear-negative TB, EPTB, and LTBI and the addition of peptides to stimulate CD8+ T cells lead to increased sensitivity of the test in detecting latent and active TB infection in patients with destroyed CD4+ T cells [24], [25], [9], [10], [11]. However, in another study, it was shown that during *M. tuberculosis* infection in the absence of CD4+ T cells, a reduced fraction of CD8+ T cells were actively producing IFN-γ *in vivo*; IFN- ɣ production is mostly derived from CD4+ T cells and CD8+ T cells contribute less to IFN- ɣ production [26].

In our study, the response to both TB1 and TB2 antigens was 91.4%, and “only to TB2” response was 2.9% in active TB patients. In comparison of PTB and EPTB, cavitary PTB and noncavitary PTB, smear-positive TB, and smear-negative TB, there was no difference between the positivity rates and the median IFN-ɣ concentration released in TB1 and TB2 tubes. However, compared to ICP, it was determined that the values of median IFN-ɣ released in both TB1 and TB2 tubes were significantly higher in NICP. In addition, although not statistically significant, the median IFN-ɣ concentration released in the TB2 tube was higher than the median IFN-ɣ concentration released in the TB1 tube in NICP, and vice versa in ICP. Although the median IFN- ɣ values were higher in TB2 than in TB1, irrespective of HIV status, there was no significant difference between positivity rates in the Zambian study [18]. Contradictory with studies that found an association between active TB and a higher IFN-γ release in TB2 compared to TB1 [25], [27]. But in concordance with Japanese and Korean studies, no significant difference was found between the positivity rates and secreted IFN-ɣ values in TB1 and TB2 tubes in active TB, in our study [19], [21].

While some authors argued that, QFT-Plus was more sensitive compared to QFT-GIT for detecting *M. tuberculosis* infection, mainly due to TB2 responses [27], [28], studies comparing the QFT-Plus and QFT-GIT tests mostly revealed equivalent sensitivity and a high overall agreement between the two tests in active TB [29], [19], [20], [21]. In our previous study, the sensitivity of QFT-GIT was also found to be 84.2% (32/38) [30].

These findings indicated that both CD4+ T cell and CD8+ T cell response was stronger in NICP than ICP, regardless of the clinical form and clinical severity of TB, but the TB2 tube added to the QFT-Plus did not bring an additional contribution to the sensitivity of the test in active TB.

Immunocompromised LTBI cases were not included in the study to avoid factors that would adversely affect the results of the QFT-Plus assay. Compared with active TB, response to both TB1 and TB2 (70.3%) was significantly lower, and response to “only to TB2” (18.8%) was significantly higher in LTBI. In addition, compared with TB, the median IFN-ɣ concentration released in both TB1 and TB2 tubes was significantly lower in LTBI. Pieterman ED et al. [31] suggested that compared with active TB, the median IFN-ɣ concentration released in both TB1 and TB2 tubes was lower in LTBI, Young Hong J.et al [21] found that patients with active TB showed higher IFN-γ concentrations to TB2 stimulation compared to individuals with LTBI.

The findings regarding the effect of TB2 tube added to the QFT-Plus assays on the sensitivity of the test in LTBI are contradictory. Petruccioli et al. [32] argued that the majority of LTBI cases simultaneously respond to both TB1 and TB2 antigens and that an “only to TB2” response is associated with active TB, whereas others reported that individuals with LTBI showed a higher CD8+ T cell response and that an “only to TB2” response is associated with LTBI [20], [25], [33]. In addition, various studies found higher CD8+ T cell responses higher IFN-γ release in TB2 in recent *M. tuberculosis* exposure compared to remote *M. tuberculosis* exposure [27], [31], [34]. Consequently, some authors suggested that the addition of the TB2 tube leads to a significant increase in sensitivity of QFT-Plus [28], [33], others found comparable results between the QFT-GIT and QFT-Plus assays in LTBI [20], [21], [31], [35].

Our findings also support that LTBI cases show a higher CD8+ T cell response and that an “only to TB2” response is associated with LTBI and that the TB2 tube may contribute to the diagnosis of LTBI. Consistent with other studies, there was also a high degree of correlation between the median IFN-ɣ values released in the QFT-Plus TB1 and TB2 tubes of LTBI cases in our study [20], [21], [35].

To our knowledge, this study is the first to evaluate the performance of the QFT-Plus and concentrations of secreted IFN-ɣ in different *M. tuberculosis* clones. There was no difference in QFT-Plus positivity rates and secreted IFN-ɣ concentrations between different *M. tuberculosis* clones. However, IFN-ɣ concentrations secreted from TB1 tubes and TB2 tubes were higher in patients infected with *M. tuberculosis*, compared with patients infected with zoonotic strains (*M. bovis, M. caprae*), but the difference was not statistically significant. This may be because the human immune system developed a stronger cellular immune response against *M. tuberculosis* in the process of co-evolution of the host pathogen that developed over thousands of years. It is necessary to conduct more detailed studies in this regard.

The most important limitation of this study was the low number of cases. In the presented study, it was concluded that, (i) although the TB2 tube contains modified peptides optimized to activate *M. tuberculosis*-specific CD8+ T cell, there was no difference between the median IFN-ɣ values released in the TB1 and TB2 tubes in both active TB and LTBI, (ii) compared to active TB, individuals with LTBI had lower T cell response and lower IFN-ɣ concentrations (iii) the positivity rate and secreted IFN-ɣ concentrations in the TB2 tube were not higher in PTB, cavitary TB, and smear-positive TB, (iv) immunocompromisation reduced secreted IFN-ɣ concentrations and sensitivity of the test and increased indeterminate results, (v) there was no difference in secreted IFN-ɣ concentrations between *M. tuberculosis* clones, but higher T cell response and IFN-ɣ concentrations in patients infected with *M. tuberculosis* strains compared to patients infected with zoonotic strains, and (vi) TB2 tube increased sensitivity in LTBI but did not contribute to sensitivity in active TB.

## Ethical approval

Ethical approval was obtained for the study from Ege University Faculty of Medicine Medical Research Ethical Committee dated 16/04/2020-E108132.
